# Associations of multiple visual rating scales based on structural magnetic resonance imaging with disease severity and cerebrospinal fluid biomarkers in patients with Alzheimer’s disease

**DOI:** 10.3389/fnagi.2022.906519

**Published:** 2022-07-29

**Authors:** Mei-dan Wan, Hui Liu, Xi-xi Liu, Wei-wei Zhang, Xue-wen Xiao, Si-zhe Zhang, Ya-ling Jiang, Hui Zhou, Xin-xin Liao, Ya-fang Zhou, Bei-sha Tang, Jun-Ling Wang, Ji-feng Guo, Bin Jiao, Lu Shen

**Affiliations:** ^1^Department of Neurology, Xiangya Hospital, Central South University, Changsha, China; ^2^Department of Radiology, Xiangya Hospital, Central South University, Changsha, China; ^3^National Clinical Research Center for Geriatric Disorders, Central South University, Changsha, China; ^4^Key Laboratory of Hunan Province in Neurodegenerative Disorders, Central South University, Changsha, China; ^5^Department of Geriatrics, Xiangya Hospital, Central South University, Changsha, China; ^6^Engineering Research Center of Hunan Province in Cognitive Impairment Disorders, Central South University, Changsha, China; ^7^Hunan International Scientific and Technological Cooperation Base of Neurodegenerative and Neurogenetic Diseases, Changsha, China; ^8^Key Laboratory of Organ Injury, Aging and Regenerative Medicine of Hunan Province, Changsha, China

**Keywords:** Alzheimer’s disease, visual rating scale, medial temporal lobe atrophy, posterior atrophy, global cerebral frontal atrophy, white matter lesions, cerebrospinal fluid

## Abstract

The relationships between multiple visual rating scales based on structural magnetic resonance imaging (sMRI) with disease severity and cerebrospinal fluid (CSF) biomarkers in patients with Alzheimer’s disease (AD) were ambiguous. In this study, a total of 438 patients with clinically diagnosed AD were recruited. All participants underwent brain sMRI scan, and medial temporal lobe atrophy (MTA), posterior atrophy (PA), global cerebral atrophy-frontal sub-scale (GCA-F), and Fazekas rating scores were visually evaluated. Meanwhile, disease severity was assessed by neuropsychological tests such as the Mini-Mental State Examination (MMSE), Montreal Cognitive Assessment (MoCA), and Clinical Dementia Rating (CDR). Among them, 95 patients were tested for CSF core biomarkers, including Aβ_1–42_, Aβ_1–40_, Aβ_1–42/_Aβ_1–40_, p-tau, and t-tau. As a result, the GCA-F and Fazekas scales showed positively significant correlations with onset age (*r* = 0.181, *p* < 0.001; *r* = 0.411, *p* < 0.001, respectively). Patients with late-onset AD (LOAD) showed higher GCA-F and Fazekas scores (*p* < 0.001, *p* < 0.001). With regard to the disease duration, the MTA and GCA-F were positively correlated (*r* = 0.137, *p* < 0.05; *r* = 0.106, *p* < 0.05, respectively). In terms of disease severity, a positively significant association emerged between disease severity and the MTA, PA GCA-F, and Fazekas scores (*p* < 0.001, *p* < 0.001, *p* < 0.001, *p* < 0.05, respectively). Moreover, after adjusting for age, gender, and *APOE* alleles, the MTA scale contributed to moderate to severe AD in statistical significance independently by multivariate logistic regression analysis (*p* < 0.05). The model combining visual rating scales, age, gender, and *APOE* alleles showed the best performance for the prediction of moderate to severe AD significantly (AUC = 0.712, sensitivity = 51.5%, specificity = 84.6%). In addition, we observed that the MTA and Fazekas scores were associated with a lower concentration of Aβ_1–42_ (*p* < 0.031, *p* < 0.022, respectively). In summary, we systematically analyzed the benefits of multiple visual rating scales in predicting the clinical status of AD. The visual rating scales combined with age, gender, and *APOE* alleles showed best performance in predicting the severity of AD. MRI biomarkers in combination with CSF biomarkers can be used in clinical practice.

## Introduction

Alzheimer’s disease (AD) is the main type of dementia, which not only causes huge social and economic burden but also seriously affect the health and quality of life of patients (Pimenova et al., 2018). Early diagnosis of AD is critical to decelerating the progress of the disease (Avila and Perry, 2021; Wan et al., 2022). Currently, the definitive diagnosis of AD relies mostly on CSF core biomarker analysis, positron emission tomography (PET) of tracer molecules, and neuropsychological tests. However, the high invasiveness of CSF and expensive costs of PET limit their wide application in clinical practice (Bateman et al., 2020; Khan et al., 2020). As a non-invasive means of AD diagnosis, structural magnetic resonance imaging (sMRI) is constantly used for early diagnosis and severity evaluation of AD (Fan et al., 2022; Zhang et al., 2022). For example, MRI is used to show patterns of brain damage and differentiate AD from other brain diseases (Chandra et al., 2019).

Alzheimer’s disease patients are found to have severe atrophy compared with cognitively normal subjects, and the atrophy degree increases with AD pathological alteration (Schwarz et al., 2016; Mårtensson et al., 2020). AD brain atrophy was mainly reported as medial temporal and parietal atrophy patterns (Kaye et al., 2005; Caspers et al., 2021). Thus, multiple visual rating scales, including medial temporal lobe atrophy (MTA), posterior atrophy (PA), global cerebral atrophy-frontal sub-scale (GCA-F), and Fazekas, play important roles in evaluating different brain variations and atrophy with AD (Harper et al., 2015, 2016; Rhodius-Meester et al., 2017). The MTA scale was put forward by Scheltens to evaluate in 1992 (Scheltens et al., 1992). Koedam et al. (2011) put forward PA in 2011, focusing on the structural changes of the posterior cingulate sulcus, parieto-occipital sulcus, precuneus, and parietal cortex. The GCA-F has been gradually applied to distinguish AD and other dementia (Scheltens et al., 1997; Zhu et al., 2021). AD patients were proved to have more severe whole white matter lesions (WMLs) than cognitively normal subjects, which could be evaluated by the Fazekas scale (Fazekas et al., 1987; McAleese et al., 2017; Zhu et al., 2021). Some previous studies used visual rating scales to build a model for predicting and diagnosing AD, identifying patients with AD from normal aging (Sheng et al., 2020), or distinguishing early- and late-onset AD (Yuan et al., 2019; Park et al., 2021; Silhan et al., 2021). Mao et al. (2021) reported that MTA was positively correlated with the age of patients with AD, rather than PA in a cohort of 112 AD patients; however, they did not report a correlation between visual rating scales and disease severity. There are no studies yet conducted to identify which of these scales have a significant advantage in detecting onset age and disease duration and which can be the best predictor of AD severity.

Cerebrospinal fluid (CSF) biomarkers are known as diagnostic tools for AD. Classic AD CSF biomarkers are characterized by low concentrations of amyloid-β (Aβ)_1–42_ and high concentrations of phosphorylated tau181p (p-tau) and total tau (t-tau) (Olsson et al., 2016; Jack et al., 2018). CSF biomarkers are great diagnostic tools for early onset cognitive impairment, and Joachim et al. (2018) reported that mild cognitive impairment (MCI) patients with a lower CSF Aβ_1–42_ level had higher risk of progressing to dementia, and CSF t-tau is mainly correlated to the progression of neurodegeneration. Recently, combinations of sMRI and CSF biomarkers are recommended for clinical diagnosis. Previous studies showed that in patients with low concentrations of Aβ_1–42_, sMRI biomarkers such as MTA and PA could predict MCI progression to AD (Van Rossum et al., 2012; Pyun et al., 2017), and also PA was associated with high levels of t-tau and p-tau (Lehmann et al., 2013). Thus, we aim to identify which visual rating scale is most consistent with CSF core biomarkers.

The current study is to determine the correlation of multiple visual rating scales (including MTA, PA, GCA-F, and Fazekas scales) with different clinical status, severity, and CSF of AD, and whether multiple visual rating scales can predict the stage of AD in a large Chinese south cohort.

## Materials and methods

### Subjects

A total of 438 AD patients (female: 266; onset age: 62.07 ± 10.113 years; age: 64.77 ± 9.99 years; disease duration: 2.723 ± 2.220; Mini-Mental State Examination (MMSE): 13.22 ± 6.290; Montreal Cognitive Assessment (MoCA): 8.336 ± 5.258; Clinical Dementia Rating (CDR): 1.282 ± 0.542) were recruited from the Department of Neurology, Xiangya Hospital, Central South University, between 2017 and 2021. The inclusion criteria for patients were as follows: (1) significant cognitive decline; (2) accessible to conduct physical examination and neuropsychological tests; (3) brain atrophy confirmed by MRI; and (4) all patients were “probable AD” diagnosed by the National Institute of Neurological and Communicative Disorders and Stroke and the Alzheimer’s Disease and Related Disorders Association (NINCDS-ADRDA) criteria. The exclusion criteria were as follows: (1) brain organic or functional disease; (2) cannot cooperate with MRI scanning, physical examination and neuropsychological tests; and (3) hypertension, diabetes, hyperhomocysteinemia, or other systemic diseases. Among them, all participants were evaluated by two experienced neurologists using the MMSE, MoCA, and CDR. The study was approved by the Ethics Committee of Xiangya Hospital, Central South University (equivalent to an institutional review board). All participants or guardians provided written informed consent.

### Magnetic resonance imaging study

Brain magnetic resonance imaging (MRI) was obtained from a 3.0 T MRI scanner (Signa EXCITE, General Electric, Fairfield, Connecticut, United States). Volumetric (3D) T1-weighted images were acquired, with thickness/gap = 3/1 mm, echo time (TE) = 30 ms, pulse repetition time (TR) = 2,000 ms, TI = 380 ms, flip angle = 90, matrix = 64 × 64, field of view (FOV) = 220 mm × 220 mm, and voxel size = 0.5 mm × 0.5 mm × 0.5 mm. T1-weighted sequence, T1-weighted coronal thin layer scanning, T2-weighted sequence, and T2 FLAIR sequence were all collected.

#### Medial temporal lobe atrophy

The MTA scale is defined in the hippocampus, parahippocampal gyrus, and surrounding CSF spaces. It ranks the degree of MTA from 0 to 4, with grade 0 denoting no cortical atrophy; grade 1, enlargement of choroid fissure; grade 2, along with enlargement of the temporal horn of the lateral ventricle; grade 3, moderate loss of hippocampal volume; and grade 4, severe loss of hippocampal volume (Scheltens et al., 1992).

#### Posterior atrophy

The PA scale is defined in the posterior cingulate sulcus, parieto-occipital sulcus, precuneus, and parietal cortex. It ranks atrophy from 0 to 3, with grade 0 showing no cortical atrophy; grade 1, mild parietal cortical atrophy, with mild enlargement of the posterior cingulate and parieto-occipital sulcus; grade 2, substantial parietal atrophy, with substantial enlargement of the posterior cingulate and parieto-occipital sulcus; grade 3, end-stage “knife-blade” atrophy, with extreme enlargement of the posterior cingulate and parieto-occipital sulcus (Koedam et al., 2011).

#### Global cerebral atrophy-frontal sub-scale

The GCA-F is defined in the frontal cortex and sulcal. It ranks 0 to 3, with grade 0 representing no cortical atrophy; grade 1, mild atrophy (dilatation of sulci); grade 2, moderate atrophy (loss of gyri volume); and grade 3, end-stage “knife blade atrophy” (Zhu et al., 2021).

#### Fazekas scale

The Fazekas scale is defined in the whole WMLs. It ranks 0–3, with grade 0 showing no or single (max 3) punctate lesion; grade 1, multiple (≥3) punctate lesions; grade 2, beginning confluency of lesions (bridging); and grade 3, large confluent lesions (Fazekas et al., 1987).

In total, two experienced neurologists were trained on the consistency of visual rating scale evaluation; 379 MTA, 437 PA, 438 GCA-F, and 438 Fazekas rating scores were evaluated by two experienced neurologists independently, who were blinded to clinical and demographic information.

### Apolipoprotein E genotyping

The venous blood was collected from 397 patients in tubes containing ethylenediaminetetraacetic acid (EDTA). Genomic DNA was extracted by using the standard phenol-chloroform extraction method. All DNA samples were diluted to 50 ng/μl. A 581-bp fragment was amplified by the following primers: forward 5′-CCTACAAATCGGAACTGG-3′ and reverse 5′-CTCGAACCAGCTCTTGAG-3′. PCR was performed as previous methods (Jiao et al., 2014). Each PCR product was sequenced by using an ABI 3730xl DNA analyzer (ABI, Louis, MO, United States).

### Cerebrospinal fluid biomarker collection and analysis

The CSF was obtained from lumbar puncture samples, and 95 patients (female: 58; onset age: 55.82 ± 8.34 years; age: 58.56 ± 7.93 years; disease duration: 2.760 ± 2.172; MMSE: 12.76 ± 6.085; MoCA: 7.888 ± 5.129; CDR: 1.259 ± 0.560) were tested for CSF core biomarkers voluntarily. All analyses of CSF biomarkers (Aβ_1–42_, Aβ_1–40_, t-tau, and p-tau) were measured by enzyme-linked immunosorbent assay (ELISA) and performed by experienced technicians in strict accordance with the instructions of the manufacturer. Specifically, the Aβ_1–42_ level in CSF < 550 pg/ml, or Aβ_1–42/1–40_ ratio ≤0.1, is defined as positive amyloidosis; p-tau > 61 pg/ml in CSF is defined as neurofibrillary tangles; t-tau ≥ 452 pg/ml in CSF is defined as nerve cell death (Jiao et al., 2021).

### Statistical analysis

Quantitative statistics (onset age, age, disease duration, scores on the MMSE, MoCA, CDR) were described as the mean and standard deviation. Categorical statistic (gender) was described as proportions. The Mann–Whitney *U*-test and Kruskal–Wallis *H*-test were applied for non-normally distributed statistical variable comparisons, and independent-sample *t*-tests or ANOVA was applied for the normally distributed continuous variable comparisons. In addition, the chi-square tests were applied for categorical variable comparisons. The partial correlation analysis was performed for the correlation of visual rating scales and AD onset age, age, disease duration, and neuropsychological assessment scales of AD patients with age, gender, and *APOE* allele adjustment. The prediction of visual rating scales to AD severity was performed using multivariate logistic regression analysis with age, gender, and *APOE* alleles adjustment and receiver operating characteristic (ROC) curve analysis. The area under the curve (AUC) and representative optimal sensitivity and specificity were conducted to evaluate the performance of the models. The statistical significance of the difference in AUCs was analyzed by Delong’s test. The statistical analysis was achieved by SPSS version 25.0, R version 4.2.1., and GraphPad prism 8.0. *P* < 0.05 was considered significant.

## Results

### Demographics and single visual rating scale values in Alzheimer’s disease

Demographic features and MTA scores of the samples are shown in Table 1. When patients were dichotomized into four groups by MTA scores, significantly higher MTA scores were found in patients with the following status: older age (*p* < 0.05), longer disease duration (*p* < 0.001), and worse cognition (*p* < 0.001). Cognition was evaluated by using the MMSE, MoCA, and CDR; all are proved in the progression of AD. The correlation between the MTA scores and AD characteristics is shown in Table 2. MTA scores had a positive correlation with the age and disease duration, and the correlation coefficients range were 0.141 and 0.137 (*p* < 0.001, *p* < 0.05, respectively). Disease severity had a positive correlation with the MTA degree, such that patients with a lower MMSE and MoCA scores had a high MTA degree, and the correlation coefficients were −0.361 and −0.301 (*p* < 0.001, *p* < 0.001, respectively). Higher CDR scores had a high MTA degree, and the correlation coefficient was 0.372 (*p* < 0.001).

**TABLE 1:** Demographic features and MTA scores of the samples.

	MTA = 1	MTA = 2	MTA = 3	MTA = 4	*P*-value
Subjects	119	183	66	11	
**Age, years**					
Onset age	60.79 (9.318)	61.66 (10.147)	62.92 (10.453)	68.91 (8.983)^a^	0.07
Age	63.12 (9.131)	64.3 (9.941)	66.23 (10.374)	72.27 (8.867)	0.017*
Disease duration, years	2.367 (1.724)	2.673 (2.672)	3.311 (1.941)^ab^	3.364 (1.362)	<0.001*
Female, *N* (%)	85 (71.4)	112 (61.2)	33 (50)	7 (63.6)	0.035*
**Memory clinic**					
MMSE	15.75 (5.214)	12.91 (6.091)^a^	10.17 (6.463)^ab^	7.09 (4.06)^ab^	<0.001*
MoCA	9.91 (4.965)	7.99 (5.026)^a^	6.08 (5.231)^a^	4.22 (3.114)^a^	<0.001*
CDR	0.877 (0.489)	1.179 (0.631)^a^	1.543 (0.703)^ab^	1.5 (0.707)^a^	<0.001*

Values in the table represent mean (standard deviation).

MTA, medial temporal lobe atrophy.

^a^Significantly different from MTA = 0.

^b^Significantly different from MTA = 1.

*P*-values from ANOVA (Kruskal–Wallis *H*-test for abnormal distribution) or chi-square test.

*Difference between the groups was statistically significant (*p* < 0.05).

**TABLE 2:** Correlation analysis between each visual rating scale and AD status.

	MTA		PA		GCA-F		Fazekas	
	r	*p*	R	*p*	r	*p*	r	*p*
**Age**								
Onset age	0.106	0.051^ac^	0.039	0.437^ac^	0.181	<0.001^ac^	0.411	<0.001^ac^
Age	0.141	0.009^ac^	0.056	0.269^ac^	0.209	<0.001^ac^	0.426	<0.001^ac^
Disease duration	0.137	0.011^ac^	0.067	0.184^ac^	0.106	0.035^ac^	0.041	0.422^ac^
Gender (F/M)	−	0.009^bc^	−	0.427^bc^	−	0.018^bc^	−	0.145^bc^
*APOE*4 (±)	−	0.296^ab^	−	0.419^ab^	−	0.874^ab^	−	0.963^ab^
**Memory clinic**								
MMSE	−0.361	<0.001^abc^	−0.307	<0.001^abc^	−0.347	<0.001^abc^	−0.167	0.001^abc^
MoCA	−0.301	<0.001^abc^	−0.278	<0.001^abc^	−0.308	<0.001^abc^	−0.127	0.017^abc^
CDR	0.372	<0.001^abc^	0.239	<0.001^abc^	0.347	<0.001^abc^	0.088	0.095^abc^

^a^After correction of gender.

^b^After correction of age.

^c^After correction of APOE alleles.

*P* < 0.05 was statistically significant.

The demographic features and PA scale of the samples are shown in Table 3. The disease duration, MMSE, MoCA, and CDR were differently distributed among the different PA scores (*p* < 0.001, *p* < 0.001, *p* < 0.001, *p* < 0.001, respectively). From correlation analysis, disease severity also had a positive correlation with the PA score. The correlation coefficients of the MMSE and MoCA were −0.307 and −0.278, and the correlation coefficient of CDR was 0.239 (*p* < 0.001; Table 2).

**TABLE 3:** Demographic features and PA scale of the samples.

	PA = 0	PA = 1	PA = 2	PA = 3	*P*-value
Subjects	90	190	143	14	
**Age, years**					
Onset age	60.66 (8.861)	62.63 (10.663)	62.77 (10.163)	57.50 (7.743)	0.121
Age	62.67 (8.939)	65.64 (10.344)	65.41 (10.105)	61.07 (8.090)	0.061
Disease duration, years	2.061 (1.4646)	3.025 (2.7743)^a^	2.668 (1.6531)^a^	3.571 (1.9499)^a^	<0.001 *
Female, *N* (%)	66 (73.3)	108 (56.8)	81 (56.6)	11 (78.6)	0.01*
**Memory clinic**					
MMSE	16.11 (5.683)	13.93 (6.391)	11.49 (6.450)^ab^	7.07 (4.891)^ab^	<0.001*
MoCA	10.01 (4.956)	8.79 (5.229)	7.33 (5.101)^a^	2.15 (2.193)^abc^	<0.001*
CDR	0.896 (0.4495)	1.120 (0.6476)	1.298 (0.6816)^a^	1.654 (0.6887)^ab^	<0.001*

Values in the table represent mean (standard deviation).

PA, posterior atrophy.

^a^Significantly different from PA = 0.

^b^Significantly different from PA = 1.

^c^Significantly different from PA = 2.

*P*-values from ANOVA (Kruskal–Wallis *H*-test for abnormal distribution) or chi-square test.

*Difference between the groups was statistically significant (*p* < 0.05).

The demographic features and GCA-F scale of the samples are shown in Table 4. Patients with severe GCA-F scores showed later onset age (*p* < 0.001) and older age (*p* < 0.001), as well as longer disease duration significantly (*p* < 0.01). Patients with severe frontal cerebral atrophy underwent severe AD, which was performed in the MMSE, MoCA, and CDR significantly (*p* < 0.001, *p* < 0.001, *p* < 0.01, respectively). Significant associations were observed between GCA-F scores and onset age, and age and disease duration. The correlation coefficient range was 0.106∼0.209 and in statistical significance (*p* < 0.001, *p* < 0.001, *p* < 0.05, respectively). The gender of patients showed a significant correlation with global cerebral-frontal atrophy, and male gender was in close correlation with severe atrophy (*p* < 0.01). AD severity had a positive correlation with GCA scores. The correlation coefficients of the MMSE and MoCA were −0.347 and −0.308, and the correlation coefficient of CDR was 0.347 (*p* < 0.001; Table 2).

**TABLE 4:** Demographic features and GCA-F scale of the samples.

	GCA-F = 0	GCA-F = 1	GCA-F = 2	*P*-value
Subjects	126	199	113	
**Age, years**				
Onset age	59.16 (9.152)	62.68 (10.150)^a^	64.26 (10.413)^a^	<0.001*
Age	61.37 (8.877)	65.51 (9.945)^a^	67.27 (10.344)^a^	<0.001*
Disease duration, years	2.251 (1.6651)	2.850 (2.5982)^a^	3.027 (1.9558)^a^	0.001*
Female, *N* (%)	91 (72.2)	117 (58.8)^a^	59 (52.2)^b^	0.005*
**Memory clinic**				
MMSE	15.69 (5.470)	13.30 (6.224)^a^	10.36 (6.109)^ab^	<0.001*
MoCA	9.87 (5.215)	8.60 (5.176)	6.09 (4.714)^ab^	<0.001*
CDR	0.845 (0.4581)	1.160 (0.6449)^a^	1.448 (0.6759)^ab^	0.001*

Values in the table represent mean (standard deviation).

GCA, global cerebral atrophy-frontal sub-scale.

^a^Significantly different from GCA = 0.

^b^Significantly different from GCA = 1.

*P*-values from ANOVA (Kruskal–Wallis *H*-test for abnormal distribution) or chi-square test.

*Difference between the groups was statistically significant (*p* < 0.05).

Demographic features and Fazekas of the samples are shown in Table 5. Patients with deteriorative WMLs showed later onset age (*p* < 0.001) and older age (*p* < 0.001) in statistical significance. Patients with higher Fazekas scores may undergo severe AD. A significant positively correlation was found between the Fazekas score and onset age and age (*p* < 0.001, *p* < 0.001). The correlation coefficients were 0.411 and 0.426. AD severity had a mild positive correlation with WMLs by the partial correlation analysis, and the correlation coefficients of the MMSE and MoCA were −0.167 and −0.127 (*p* < 0.01, *p* < 0.05, respectively; Table 2).

**TABLE 5:** Demographic features and Fazekas of the samples.

	Fazekas = 0	Fazekas = 1	Fazekas = 2	Fazekas = 3	*P*-value
Subjects	21	264	121	32	
**Age, years**					
Onset age	56.62 (10.745)	59.28 (9.026)	66.82 (9.954)^ab^	70.78 (6.298)^ab^	<0.001*
Age	59.29 (10.233)	61.85 (8.875)	69.78 (9.72)^ab^	73.53 (6.17)^ab^	<0.001*
Disease duration, years	2.667 (1.5599)	2.601 (1.8368)	2.988 (3.0018)	2.766 (1.9959)	0.807
Female, *N* (%)	12 (57.1)	173 (65.5)	67 (55.4)	15 (46.9)	0.802
**Memory clinic**					
MMSE	15.05 (5.509)	13.48 (6.152)	12.71 (6.401)	11.72 (7.217)	0.235
MoCA	10.29 (4.660)	8.16 (5.228)	8.5 (5.062)	7.77 (6.550)	0.227
CDR	0.952 (0.4976)	1.126 (0.6099)	1.186 (0.7227)	1.339 (0.6878)	0.236

Values in the table represent mean (standard deviation).

^a^Significantly different from Fazekas = 0.

^b^Significantly different from Fazekas = 1.

*P*-values from ANOVA (Kruskal–Wallis *H*-test for abnormal distribution) or chi-square test.

*Difference between the groups was statistically significant (*p* < 0.05).

The 438 AD patients were divided into two groups according to the onset age: disease started before the age of 65 years was early onset AD (EOAD) and the other was late-onset AD (LOAD). Table 6 shows the characteristics of patients with EOAD compared to patients with LOAD. Patients with EOAD had worse cognitive function as measured by the MMSE and MoCA (*p* < 0.01, *p* < 0.001). In addition, patients with LOAD showed severe global cerebral atrophy and WMLs (*p* < 0.001, *p* < 0.001). The levels of CSF biomarkers were comparable among groups.

**TABLE 6:** Characteristics of AD patients according to age at onset.

	EOAD	LOAD	*P*-value
Subjects	257	181	
**Age, years**			
Onset age	54.93 (5.575)	72.22 (5.254)	<0.001*
Age	57.78 (5.667)	74.70 (5.300)	<0.001*
Female, *N* (%)	167 (65.0)	100 (55.2)	0.04*
*APOE*4 carriers (%)	41.4	46.1	0.354
**Memory clinic**			
MMSE	12.45 (6.348)	14.32 (6.054)	0.002*
MoCA	7.56 (5.244)	9.43 (5.094)	0.634
CDR	1.163 (0.6498)	1.132 (0.6498)	<0.001*
**sMRI score**			
MTA	1.86 (0.749)	2.00 (0.806)	0.092
PA	1.16 (0.821)	1.23 (0.752)	0.362
GCA-F	0.85 (0.753)	1.14 (0.684)	<0.001*
Fazekas	1.17 (0.554)	1.66 (0.762)	<0.001*
**CSF biomarkers**			
Aβ42 (pg/mL)	481.08 (270.47)	417.65 (174.52)	0.346
Aβ40 (pg/mL)	8330.16 (4503.81)	8024.47 (5029.87)	0.831
Aβ42/Aβ40	0.06 (0.29)	0.049 (0.26)	0.101
P-Tau (pg/mL)	104.51 (51.29)	104.85 (47.70)	0.98
T-Tau (pg/mL)	497.64 (304.36)	453.13 (335.59)	0.585

Values in the table represent mean (standard deviation). Visual rating scales scores of AD patients according to age at onset (*n* = 438). CSF biomarkers of AD patients according to age-at-onset (*n* = 95). *P*-values from t-test or chi-square test.

*Difference between the groups was statistically significant (*p* < 0.05).

### The value of visual rating scales in the prediction of Alzheimer’s disease severity

**FIGURE 1:**
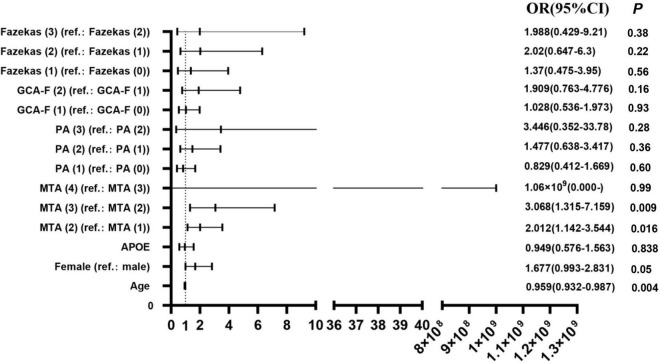
Regression analyses of visual rating scales in the prediction of AD severity (*N* = 435). OR, odds ratio; 95% CI, 95% confidence interval; B, regression coefficient. *P* < 0.05 was statistically significant.

**FIGURE 2:**
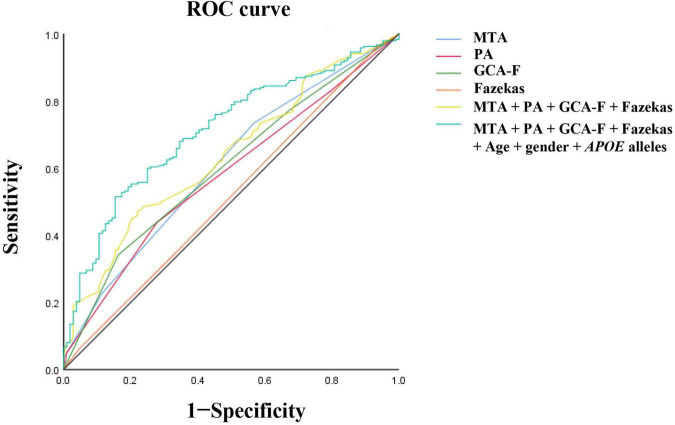
Receiver operating characteristic (ROC) curve analysis of visual rating scales for disease severity. The MTA exhibited the best predictive efficacy as a single indicator (AUC = 0.622, sensitivity = 74%, specificity = 44.6%). The PA, AUC = 0.59, sensitivity = 44.2%, specificity = 73.2%. The GCA-F, AUC = 0.609, sensitivity = 34.7%, specificity = 84.8%. The Fazekas, AUC = 0.51, sensitivity = 95.5%, specificity = 6.2%. The MTA combined with PA, GCA-F, and Fazekas; the predictive performance significantly improved (AUC = 0.654, sensitivity = 48.3%, specificity = 78.6%). The predictive model combining the MTA, PA, GCA-F, Fazekas, age, gender, and *APOE* alleles showed the best performance for the identification of moderate to severe AD significantly (AUC = 0.712, sensitivity = 51.5%, specificity = 84.6%). The Delong test was used to compare the difference of predictive models. AUC, area under the curve; ROC, receiver operating characteristic curve.

The participants with AD were divided into two groups (133 mild AD patients, 302 moderate to severe AD patients) according to the MMSE scores. Mild AD was 18 points ≤ MMSE ≤ 23 points and moderate to severe AD was MMSE < 18 points (Yuan et al., 2019). The average age of mild AD patients was 66.26 ± 10.314, and the average age of moderate to severe AD patients was 64.12 ± 9.842. The proportion of female patients with mild AD was 54.1, and the proportion of female patients in moderate to severe AD was 64.2. We used multivariate logistic regression analysis to explore the value of rating scales in the prediction of the severity of AD, after adjusting for age, gender, and *APOE* alleles (Figure 1). Logistic regression analysis showed that female gender contributed statistical significance to the discrimination between mild and moderate to severe AD (*B* = 0.577, *p* < 0.05). The MTA scale was the only scale that contributed to moderate to severe AD in statistical significance independently (*p* < 0.05). Moreover, ROC curves were generated to evaluate the performance of the visual rating scales to predict the moderate to severe AD from mild AD (Figure 2). As a single scale, MTA showed best predictive efficacy (AUC = 0.622, sensitivity = 74%, specificity = 44.6%). After incorporating age, gender, *APOE* alleles, PA, GCA-F, and Fazekas into the model, the model showed best performance for the prediction of moderate to severe AD (AUC = 0.712, sensitivity = 51.5%, specificity = 84.6%).

### Visual rating scales and cerebrospinal fluid biomarkers

**FIGURE 3:**
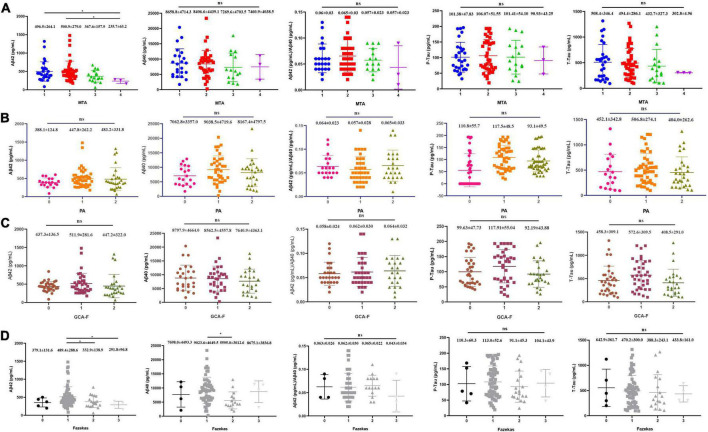
Each visual rating scale and correlation of CSF core biomarkers. **(A)** MTA (*N* = 92), **(B)** PA (*N* = 95), **(C)** GCA-F (*N* = 95), and **(D)** Fazekas (*N* = 95). *P*-values from ANOVA (Kruskal–Wallis *H*-test for abnormal distribution), and Games–Howell test for *post-hoc* comparison. *P < 0.05 was statistically significant; ns: no statistical difference.

The relationships between visual rating scales including MTA, PA, GCA-F, Fazekas, and CSF biomarkers were shown in Figure 3. A lower concentration of Aβ_1–42_ was significantly associated with MTA. AD patients with MTA = 4 (235.65 ± 63.15 pg/mL) presented a significantly lower level of Aβ_1–42_ than patients with MTA = 1 (496.48 ± 264.08 pg/mL, *p* < 0.05) and MTA = 2 (500.93 ± 279.00 pg/mL, *p* < 0.05). White matter lesions were significantly associated with lower concentrations of Aβ_1–42_ and Aβ_1–40_. Compared to Fazekas = 1 (489.43 ± 288.59 pg/mL), the average levels of Aβ_1–42_ in Fazekas = 2 and Fazekas = 3 (332.91 ± 138.89 pg/mL and 291.80 ± 96.84 pg/mL, *p* < 0.05, *p* < 0.05, respectively) were lower. Compared to Fazekas = 1 (9023.63 ± 4649.50 pg/mL), the average level of Aβ_1–40_ in Fazekas = 2 (5594.98 ± 3012.56 pg/mL, *p* < 0.05) was lower. However, the remnant visual rating scales were not associated with the rest of CSF biomarkers, such as p-tau and t-tau (Jiao et al., 2021).

## Discussion

We systematically analyzed the benefits of multiple visual rating scales in presenting AD clinical status and disease severity in a Chinese south cohort. We found that visual rating scales played significant roles in presenting the AD status, including onset age, disease duration, and severity of AD, and different scales showed individual performance. To our knowledge, the MTA, GCA-F, and Fazekas were positively correlated with age at onset and age at diagnosis, and the Fazekas showed best performance. Previous studies showed neurodegenerative diseases like AD were performed with different patterns of brain atrophy (Scheltens et al., 2002; Silhan et al., 2020), and the different atrophy subtypes were with significantly different demographic and clinical characteristics (Poulakis et al., 2018). Claus et al. (2017) proposed the MTA scale strongly increased with age in a memory clinic in which only the MTA scale was conducted. In addition, small cohorts showed elderly AD patients had pronounced hippocampal atrophy, and early onset AD patients were more associated with generalized atrophy (Schoonenboom et al., 2008; Sluimer et al., 2008). Kao et al. (2019) suggested that age was significantly associated with WMLs. Most of the previous studies applied only a single visual rating scale, while we provided information on multiple visual rating scales with the onset age in a large Chinese south AD cohort. Thus, our study is more meaningful, although our findings might be inconsistent with them. Our observation of sMRI rating scales in patients with EOAD and LOAD showed that patients with LOAD scored higher on GCA-F and Fazekas. In terms of disease duration, a positive significant association emerged between disease duration with MTA and GCA-F, especially with MTA. We revealed the effect of disease duration on the integrity of the brain. Pyun et al. (2017) noted that women had an increased risk of brain atrophy (Ferretti et al., 2018; Sapkota et al., 2021), but our results showed men had a higher proportion of worse global cerebral frontal atrophy and MTA. This might be because brain atrophy accelerates over time, and many factors such as hypertension, hyperlipidemia, smoking, and drinking result in small vessel disease, which may lead to brain atrophy, while these factors occur mostly in men. Besides, male may perform relatively elder metabolic brain age in adulthood (Goyal et al., 2019). Also, it may be because the samples we enrolled were limited to AD patients.

In this study, the multivariate logistic regression analysis showed that after adjusting age, gender, and *APOE* alleles, the MTA scale showed the highest performance in the prediction of disease severity, compared with PA, GCA-F, and Fazekas. It was in accordance with some previous studies. The MTA was significantly associated with lower cognition evaluated by MMSE scores and showed the best diagnostic value and predictive performance compared to the GCA-F and PA scales in the Alzheimer’s Disease Neuroimaging Initiative (ADNI) cohort (Geroldi et al., 2006; Perneczky et al., 2009; Ferreira et al., 2015; Wei et al., 2019). Yuan et al. (2019) showed the medial temporal lobe and anterior cingulate atrophy were negatively correlated with the scores of the MMSE in a small AD cohort. In addition, Hett et al. (2021) presented a framework to apply the multiscale graph-based grading method to analyze the alterations in whole-brain structures. They found the most prominent zones were the post-central gyrus, anterior cingulate gyrus, hippocampus, and precuneus and proved it was a competitive tool to predict AD progression in the ADNI dataset (Hett et al., 2021). However, their method depended on the quality of graph segmentations and computer-aided diagnostic systems, thereby increasing the complexity of application for clinicians. Thus, the MTA remains the most typical imaging biomarker to predict the progression and severity of AD, and the visual rating scale is an easier and faster option for clinical practice. To our knowledge, both PA and GCA scales are also positively correlated with clinical cognition, following the MTA scale. Claus et al. (2017) and Pyun et al. (2017) showed PA was associated with rapid progression of AD, and the GCA-F was significantly associated with worse clinical cognition with lower MMSE and CDR scores (Stenset et al., 2008). Interestingly, the model combining multiple visual rating scales, age, gender, and *APOE* alleles showed the best performance for the predicting moderate to severe AD. It was feasible and necessary to systematically analyze multiple visual rating scales and incorporate the multiple scales into clinical practice. Within this study, the MMSE, MoCA, and CDR were combined to synthetically evaluate the severity of AD, for the first time. We found that all of them were significantly associated with visual rating scales, while in many previous studies, only the MMSE or CDR was applied to evaluate the disease severity.

Cerebrospinal fluid core biomarkers such as Aβ and tau were associated with a cognitive decline and had little change over time (Li et al., 2016; Bos et al., 2017). As brain atrophy progresses with disease advancement, the rate of atrophy could be considered a marker for disease progression (Rusinek et al., 2004). No studies before systematically evaluated the correlation between multiple visual rating scales and CSF biomarkers in the same population. Previous studies mainly focused on the association between CSF biomarkers and the MTA and PA in European cohorts. For example, Contador J proposed that CSF Aβ_1–42_ was correlated with faster cortical thinning, and some research studies also showed that the MTA scale was in correlation with a lower level of Aβ (Fjell et al., 2008; Schuff et al., 2009; Lehmann et al., 2013). Moreover, Berron et al. (2020) also found functional association between the medial temporal lobe and regions in the anterior–temporal system was impaired in Aβ-positive individuals. However, most of them were studied in a small cohort. Our results supported a lower concentration of Aβ_1–42_ in MTA in the Chinese south population. In addition, a significant association was found between the lower concentrations of Aβ_1–42_ and WMLs in our study, which might imply a link between white matter macrostructural and amyloid pathology microstructural damage (Pietroboni et al., 2018). Although PA was found to be associated with an increased concentration of p-tau181 in AD in previous studies (Lehmann et al., 2013; Mao et al., 2021), we did not find any association between CSF tau and brain atrophy possibly because CSF tau was a biomarker of global neurodegeneration, whereas visual rating scales were markers of local neurodegeneration (Abdelnour et al., 2020). Furthermore, the employment of multiple visual rating scales to correlate the CSF core biomarkers with different regional brain atrophy might be a useful predictive tool for clinicians to use in clinical practice.

In conclusion, compared with PA, GCA-F, and Fazekas scales, the MTA scale performs best in predicting the severity of AD, whereas the visual rating scales combined with age, gender, and *APOE* alleles showed the best performance in predicting the severity of AD. AD patients with severe global cerebral atrophy and WML load were prone to present at a later onset age, and CSF biomarkers had no association with onset age. The combination of MRI biomarkers and CSF core biomarkers can be recommended for clinical practice.

## Data availability statement

The raw data supporting the conclusions of this article will be made available by the authors, without undue reservation.

## Ethics statement

The studies involving human participants were reviewed and approved by the Ethics Committee of Xiangya Hospital, Central South University. The patients/participants provided their written informed consent to participate in this study.

## Author contributions

M-DW, BJ, and LS were responsible for the study design, acquisition of data, analysis of data, and drafting/revising the manuscript. HL, X-XLiu, W-WZ, X-WX, S-ZZ, Y-IJ, HZ, X-XLia, Y-FZ, B-ST, J-LW, and J-fG collected the data and revised the manuscript. All authors agreed to the submitted manuscript.
